# Making Every Step Count: Minute-by-Minute Characterization of Step Counts Augments Remote Activity Monitoring in People With Multiple Sclerosis

**DOI:** 10.3389/fneur.2022.860008

**Published:** 2022-05-23

**Authors:** Valerie J. Block, Matthew Waliman, Zhendong Xie, Amit Akula, Riley Bove, Mark J. Pletcher, Gregory M. Marcus, Jeffrey E. Olgin, Bruce A. C. Cree, Jeffrey M. Gelfand, Roland G. Henry

**Affiliations:** ^1^Department of Neurology, University of California San Francisco (UCSF) Weill Institute for Neurosciences, University of California, San Francisco, San Francisco, CA, United States; ^2^Department of Epidemiology and Biostatistics, University of California, San Francisco, San Francisco, CA, United States; ^3^Department of Medicine, University of California, San Francisco, San Francisco, CA, United States; ^4^Department of Radiology, University of California, San Francisco, San Francisco, CA, United States

**Keywords:** multiple sclerosis, Fitbit, remote monitoring, activity level, accelerometry, minute-by-minute steps

## Abstract

**Background:**

Ambulatory disability is common in people with multiple sclerosis (MS). Remote monitoring using average daily step count (STEPS) can assess physical activity (activity) and disability in MS. STEPS correlates with conventional metrics such as the expanded disability status scale (Expanded Disability Status Scale; EDSS), Timed-25 Foot walk (T25FW) and timed up and go (TUG). However, while STEPS as a summative measure characterizes the number of steps taken over a day, it does not reflect variability and intensity of activity.

**Objectives:**

Novel analytical methods were developed to describe how individuals spends time in various activity levels (e.g., continuous low versus short bouts of high) and the proportion of time spent at each activity level.

**Methods:**

94 people with MS spanning the range of ambulatory impairment (unaffected to requiring bilateral assistance) were recruited into FITriMS study and asked to wear a Fitbit continuously for 1-year. Parametric distributions were fit to minute-by-minute step data. Adjusted R^2^ values for regressions between distributional fit parameters and STEPS with EDSS, TUG, T25FW and the patient-reported 12-item MS Walking scale (MSWS-12) were calculated over the first 4-weeks, adjusting for sex, age and disease duration.

**Results:**

Distributional fits determined that the best statistically-valid model across all subjects was a 3-compartment Gaussian Mixture Model (GMM) that characterizes the step behavior within 3 levels of activity: high, moderate and low. The correlation of GMM parameters for baseline step count measures with clinical assessments was improved when compared with STEPS (adjusted R^2^ values GMM vs. STEPS: TUG: 0.536 vs. 0.419, T25FW: 0.489 vs. 0.402, MSWS-12: 0.383 vs. 0.378, EDSS: 0.557 vs. 0.465). The GMM correlated more strongly (Kruskal-Wallis: *p* = 0.0001) than STEPS and gave further information not included in STEPS.

**Conclusions:**

Individuals' step distributions follow a 3-compartment GMM that better correlates with clinic-based performance measures compared with STEPS. These data support the existence of high-moderate-low levels of activity. GMM provides an interpretable framework to better understand the association between different levels of activity and clinical metrics and allows further analysis of walking behavior that takes step distribution and proportion of time at three levels of intensity into account.

## Introduction

Ambulatory disability is one of the most common, bothersome and limiting symptoms for people living with multiple sclerosis (MS) and greatly decreasing quality of life (1, 2). Walking *capacity* is measured in the clinic using a variety of validated outcomes (e.g. Timed 25-Foot Walk [T25FW] test), however, measurement and evaluation of walking *performance* (i.e. what they actually do in daily life) may be more important to the patient and reflect actual function (3, 4).

Efforts by several groups focused on remote (real-world) monitoring of ambulatory function mostly using average daily step count (STEPS), obtained from research-based and commercially available accelerometers (5–12). In the Fitbit remote monitoring in MS (FITriMS) study, daily step counts were collected continuously for over 1-year (5, 6). The STEPS averaged over the first 30 days correlated with disability (Expanded Disability Status Score [EDSS]), clinic-based metrics (T25FW, Timed-Up and Go Test [TUG], 2-min walk test [2MWT]) and patient reported outcomes (i.e. 12-item MS Walking Scale [MSWS-12]) (5) Longitudinal analysis over 1 year demonstrated a change in STEPS over time, even when conventional measures remained stable (6). These findings suggest remote physical activity monitoring provides additional sensitivity when capturing change in performance in people with MS.

Physical activity (activity) is quantified in different ways. The STEPS summarizes the total number of steps taken during an allotted epoch (usually 1 day) but does not reflect how different ambulatory behavior results in unique or distinctive step distributions, nor does it provide information or understanding of variability and intensity of the activity. Minute-by-minute (M-M) step count data can provide more granular information on the intensity, duration and frequency of ambulatory behavior. The aims for this analysis were to: determine the best probabilistic model using M-M step data to characterize activity distribution in people with MS with a range of ambulatory disability, evaluate the statistical validity of this new outcome, and compare with STEPS and conventional disability correlates at baseline.

## Methods

### Study Procedures

The FITriMS study methods were described previously (5, 6). Briefly, adults (>18 years old) with either progressive or relapsing MS (13) were prospectively recruited from a single MS Center (University of California San Francisco; UCSF) into the FITriMS study between July 2015 and April 2016. For inclusion, participants were able walk continuously for at least 2 min, had WiFi access, experienced no relapse for the last 30 days, and were free from any musculoskeletal or cardiovascular comorbidities affecting ambulatory function (in the opinion of the study physical therapist). A range of ambulatory disability levels were block recruited to ensure a wide representation of ambulatory participants. MS disability was evaluated at study entry in the clinic using the EDSS (14), walking speed via the T25FW, (15), mobility and balance via the TUG (15), and endurance via a 2MWT (16, 17). Patient-reported impact of MS on walking, MSWS-12 questionnaires, was completed online using secure REDCap email link at study entry (18). Study personnel provided training on the maintenance and use of a Fitbit Flex for each participant. Participants were asked to wear the Fitbit as much as possible on their non-dominant wrist. Aggregated daily, and granular M-M, step count data from the Fitbit were uploaded and stored on the UCSF Eureka platform (https://info.eurekaplatform.org/). In this data set, “physical activity” refers to outcome derived from step count (daily or minute-by-minute). The study protocol was approved by the institutional review board at UCSF, and all participants provided informed consent.

### Quality Control (QC) and Data Cleaning

From the date of study entry, the first 4 week (or 28 days = “baseline”) of M-M step count data were gathered for each individual. The “baseline” was chosen for comparison with previous STEPS analysis (5). To ensure only valid days were analyzed, any day that had a total sum of < 128 steps/day was removed. In M-M data, data points with > 300 steps per minute were excluded. Days with fewer than 128 steps were previously reported as non-valid, non-wear days (5) Weeks with < 3 valid days were also excluded. Data cleaning was based on MS literature and our previous work on this data set where: 1) no clear pattern of reactivity (i.e., temporary increase in activity after initial donning -due to the knowledge of being monitored – followed by a drop in activity when novelty wears off) was observed, 2) higher correlation was found using 13 days or more of monitoring, and 3) lower reliability with monitoring epochs of 3 days (5, 19). Night-time sleep data from Fitbit has not been validated in people MS and the majority of our patients only wore the device during the day. Long epochs of zero data were indicative of non-use or sleep, therefore only non-zero M-M data was used for subsequent analysis.

### Analysis

After quality control, the cohort data were combined to include all valid participants. To determine the best probabilistic model and statistical validation, multiple statistical distributions were fit to the data and evaluated on an individual and group level. (Supplementary Table 1).

Previous visual observation of the step distribution revealed distinct ‘clustering’ of steps; therefore, mixture distributions (Gaussians) were included.

A single Gaussian distribution is characterized by two parameters, **μ** (the mean) and **σ** (the variance) that control the location and spread of the distribution, respectively. In a 3-component Gaussian Mixture Model (GMM), consists of several Gaussian distributions where each Gaussian is assigned a proportion (π) parameter, a mean (**μ**) parameter and a variance (**σ**) parameter. The proportion (π) describes how much each Gaussian contributes to the overall model.

Linear regression was used to compare the chosen model with clinical and patient-reported outcomes. The inverse of TUG and T25FW was used to transform the data and allow for normally distributed residuals for the linear regressions. Next, linear regression on the same Gaussian parameters including age, sex, MS subtype and STEPS with clinical and patient-reported metrics was performed. JMP, version Pro 16 (20) was used for the analysis and figure generation.

## Results

**Figure 1:**
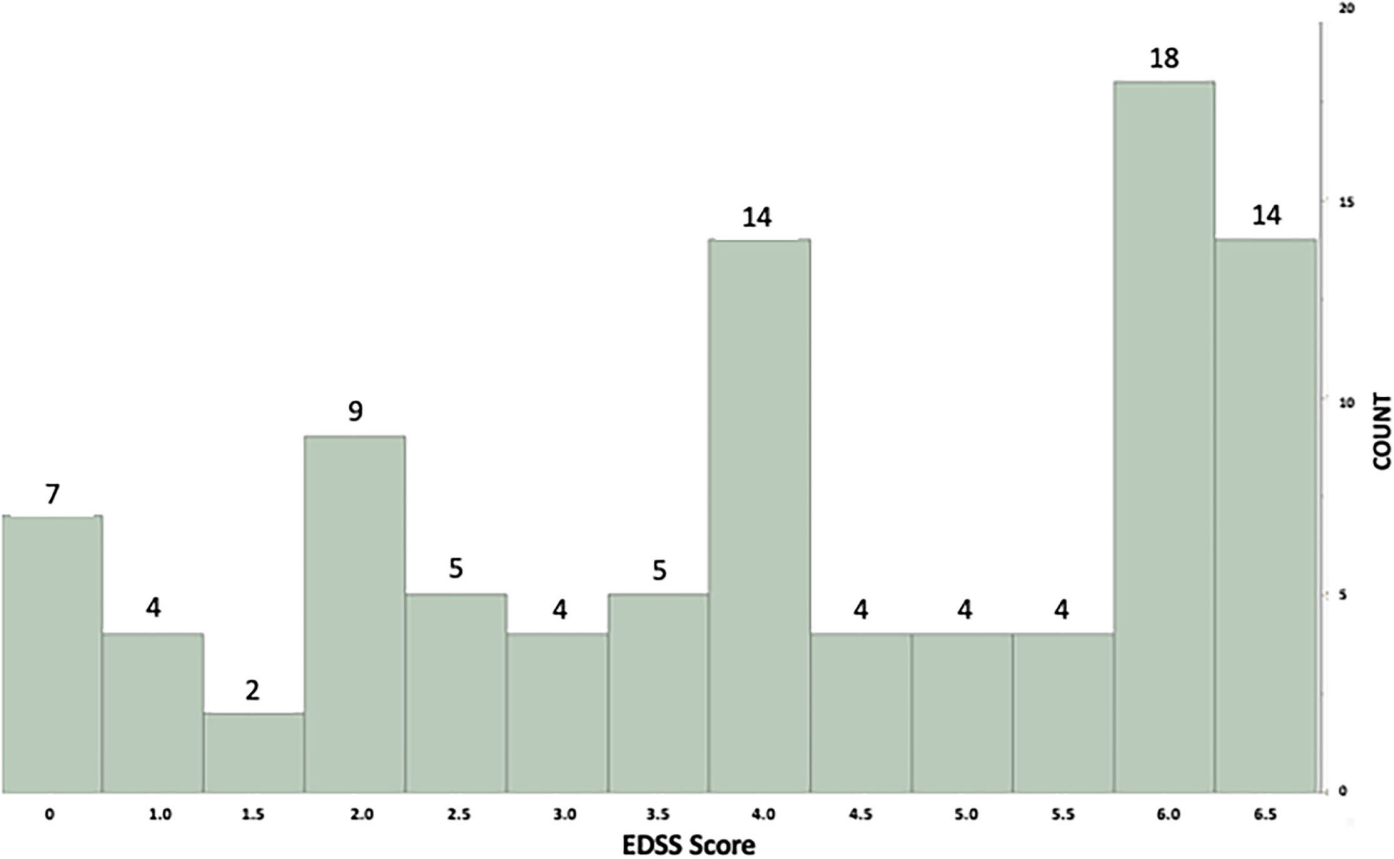
Histogram showing individual example of minute-by-minute step count distribution with the GMM model fit. X axis = Expanded Disability Status Score (EDSS) scores. Y axis = Number of subjects in each EDSS level.

From the 104 patients recruited into FITriMS, 10 did not have M-M data - due to sporadic syncing resulting in only daily step count data rather than M-M which requires weekly syncing. Of 94 participants used for this analysis, 63.5% carried a relapsing MS diagnosis (the remaining had progressive forms of MS) and more than two-thirds (76.3%) were women. The mean (SD) age was 55.5 years (13.7), median disease duration was 19.6 (IQR: 16) years, and median EDSS 4.0 (IQR: 3.5). All participant characteristics are summarized in Table 1 and EDSS distribution in Figure 1.

**Table 1:** Demographic and clinical characteristics.

	**Mean (SD)**	**Min**	**Max**	**IQR**
Age (years)	55.5 (13.7)	28	80	24.3
Number of Valid Days (Out of 28)	25.0 (5.46)	5	28	3
Step Counts (per minute)	26.5 (25.1)	1	293	25
Disease Duration (years)	19.6 (11.9)	5	55	16
TUG (seconds)	11.9 (11.3)	4.3	88.7	5.9
T25FW (seconds)	7.4 (5.6)	2.8	44.2	2.9
2MWT (meters)	133.5 (50.2)	16.5	237.6	78.1
	Median	Min	Max	IQR
EDSS	4.0	0.0	6.5	3.5
MSWS-12 (score 12–60)	41	12	60	25.5
Sex	*N* (%)	-	-	-
Male	36 (23.7)	-	-	-
Female	58 (76.3)	-	-	-
MS subtype	*N* (%)	-	-	-
Relapsing	59 (62.8)	-	-	-
Progressive	35 (37.2)	-	-	-

*EDSS, Expanded Disability Status Score; TUG, Timed-Up-and Go Test (Greater times indicate worse balance and walking ability, and higher fall risk); T25FW, Timed-25 Foot Walk test (Greater times indicate slower walking speed and greater disability); 2MWT, 2-min Walk Test (Shorter distances indicate less endurance); MSWS-12, 12-item MS Walking Scale (higher scores reflect greater self-reported impact of MS on walking). MS, multiple sclerosis; Step count, After cleaning the data; this is the average Step count per minute during active time (>0 steps/ min on valid days) - averaged over 4 weeks*.

**Figure 2:**
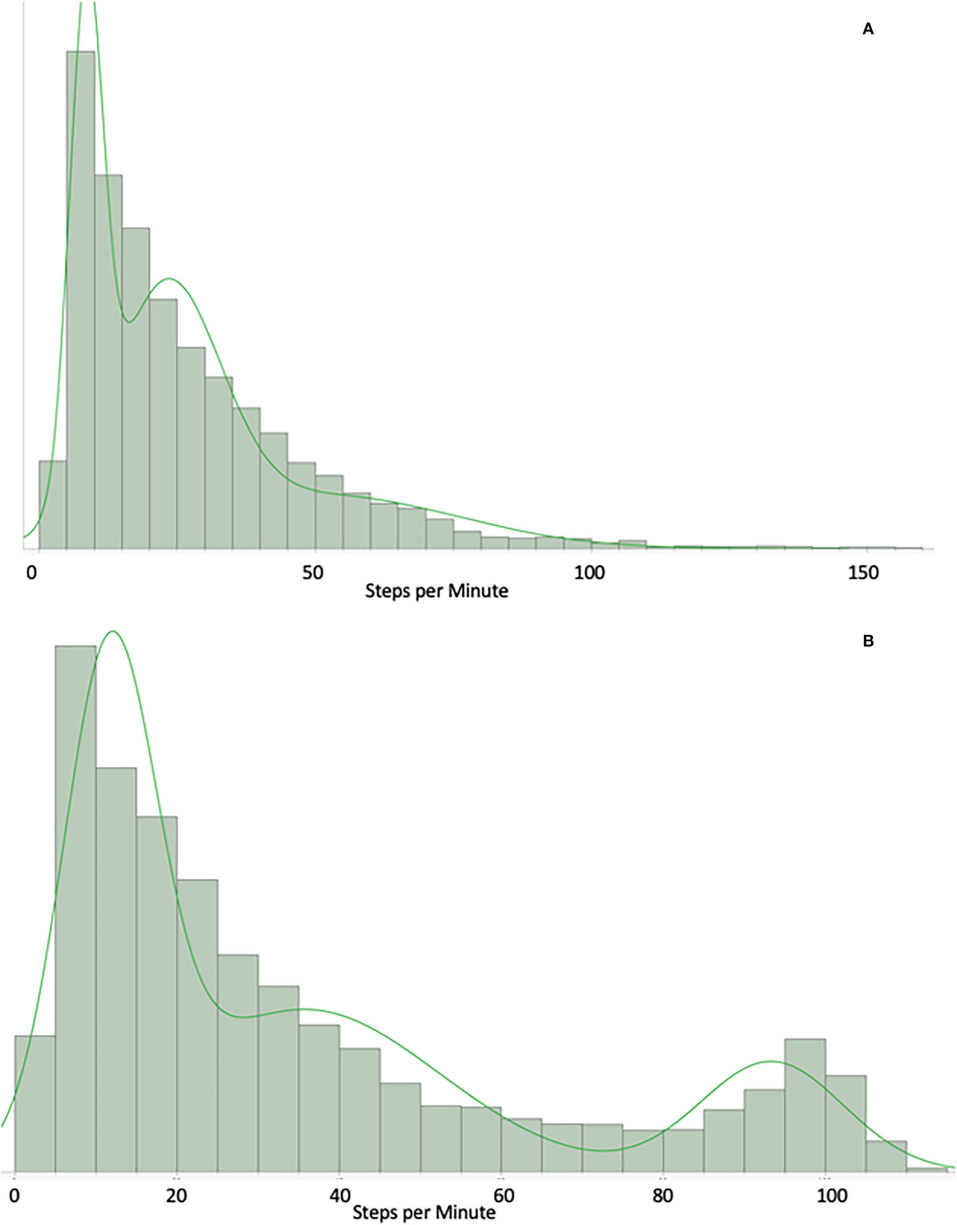
**(A)** Example of a participant who spends most of the time in low levels of physical activity. GMM: X axis = count of steps per minute over 1 month. Example of a participant who spend most of their time in low levels of physical activity (10–30 steps/min). **(B)** Example of a participant who performs some higher physical activity. GMM: X axis = count of steps per minute over 1 month. Example of a participant who do some level of higher physical activity (bump around the 100–130 steps/min).

The GMM was found to be the best fit for individual subject data (see Supplemental Table 1 for full comparison of distributions). GMM fits a greater variability in activity distribution and provides more flexibility in generalizable representation of MS activity. For example, Figure 2A shows a participant who spend most of their time in low levels of activity and Figure 2B depicts the GMM fit for a participant who perform some higher activity over the day (100–130 steps per min).

All Gaussian parameters (μ, σ, and π) except for π3 (since π1, π2 and π3 are perfectly collinear [π1 + π2 + π3 = 1]) correlated with clinical metrics (EDSS, TUG, MSWS12, 2MWT, T25FW). Using individual participant data, GMM was fit to M-M step data.

We propose that the 3 Gaussians correspond to 3 activity levels for each patient (low activity, medium activity, high activity – as ordered by their **μ** [means]): each **μ** represents the average step count we would expect from each step activity level, as characterized by its corresponding Gaussian; each **σ** represents the variability we would expect for each activity level; and each π represents the proportion of activities we would expect from each activity region.

Linear regression was used to show moderate to high correlation between GMM acquired over the study's baseline first 4 weeks of monitoring, and both clinic-based and patient-reported outcome measures. The results using GMM were consistently stronger than results obtained using only STEPS. Adding STEPS to GMM (GMM + STEPS) consistently outperforms either measure its own. Adjusting for sex, age and disease duration improved all models (Table 2).

**Table 2:** Comparison of GMM model and STEPS with conventional clinic-based and patient-reported outcomes.

**Adjusted R^**2**^**	**EDSS**	**TUG**	**T25FW**	**2MWT**	**MSWS−12**
GMM	0.557	0.536	0.489	0.560	0.383
STEPS	0.465	0.419	0.402	0.432	0.378
GMM + STEPS	0.631	0.541	0.503	0.542	0.446
GMM (Adjusted)	0.675	0.587	0.546	0.538	0.512
STEPS (Adjusted)	0.569	0.525	0.453	0.439	0.461
GMM + STEPS (Adjusted)	0.710	0.583	0.544	0.548	0.533

*EDSS, Expanded Disability Status Score; TUG, Timed-Up-and Go Test; T25FW, Timed-25 Foot Walk test; 2MWT, 2-min Walk Test; MSWS-12, 12-item MS Walking Scale. GMM - Normal 3 Mixture; STEPS, Average daily step count over the first 4 weeks (or 28 days). (Adjusted for = sex; age; and disease duration into the model)*.

Table 3A shows the centers for each Gaussian for different EDSS groupings. EDSS groups with lower levels of disability had consistently higher Gaussian centers. Further, Gaussians at higher activity levels had higher variances than those at lower levels.

**Table 3A:** Mean physical activity level distribution per disability (EDSS) group (distribution of μ).

**EDSS group [*N*]**	**Disability level**	**Low activity μ_1_ (SD)**	**Moderate activity μ_2_ (SD)**	**High activity μ_3_ (SD)**
0.0–3.5 [36]	No “walking” disability	10.96 (1.22)	32.70 (5.52)	82.37 (21.59)
4.0–5.5 [26]	Walking disability present	10.46 (1.74)	30.10 (7.65)	75.63 (27.56)
6.0 [18]	Needs a cane to ambulate	9.68 (1.20)	24.89 (4.28)	58.17 (12.94)
6.5 [14]	Needs 2 canes or a walker to ambulate	8.44 (0.94)	20.52 (2.98)	53.28 (11.51)

*Each participant was fit individually, and the mean physical activity was extracted for the group distribution. People with lower levels of disability (i.e. EDSS = 0.0–3.5) tended to have greater mean physical activity in each level (low: **μ_1_**, moderate: **μ_2_** and high: **μ_3_**)*.

## Discussion

These results provide preliminary evidence for the use of a GMM probabilistic model to characterize activity distribution using granular M-M step count in people with MS. This model performs better (stronger correlations and adjusted R^2^) than previous methods using crude STEPS.

The GMM model was able to generalize over a range of activity profiles. More specifically, it was able to capture the step distributions of those in the cohort where a significant percentage of steps come from high activity levels. Previous work from our group illustrated the wide variability in activity levels in people with MS, within and between all ambulatory disability levels (5). Therefore, the ability to generalize M-M modeling to highly variable distributions using the 3 compartment GMM has clinical appeal. In addition, the GMM dovetails well with existing literature regarding activity levels classified into three levels: low, moderate/moderate to vigorous, and high physical activity (21–23).

The GMM representation of activity outperforms other statistical models and performs better than STEPS as compared to conventional disability correlates (5). The GMM may provide an interpretable framework to better understand the association between different levels of activity and clinical metrics. It also allows further analysis of walking performance and behavior by taking step distribution and proportion of time at each intensity into account. GMM and STEPS are complimentary; STEPS provides a mean, whereas GMM presents information regarding intensity, variance, and proportional step distribution. The model including STEPS and GMM generated high correlation with the conventional outcomes, suggesting that the overall mean (STEPS) is a useful metric in combination with the more descriptive GMM.

This analysis has important limitations. Although this cohort was well-phenotyped, larger studies in more heterogeneous populations are needed to provide additional evidence of generalizability and replicability. Due to the limited availability of M-M, longitudinal (>7 days) datasets in people with MS, we were not able to perform a replication analysis. In addition, analysis of larger datasets collected in randomly recruited cohorts (rather than block enrolled) will be required. Data processed with the same granularity (minute-by-minute steps) from healthy age-matched controls would provide a better understanding about the proportions of time spent in each activity level.

A GMM based model is also relatively inflexible when approximating activity distributions that are not Gaussian in nature. A possible solution to overcome this representational limitation is to use an autoencoder (24), a type of neural network, to compress the distribution into a flexible lower dimensional representation with greater generalizability.

Without access to a platform that automatically pulls the M-M data, retrieving these detailed metrics would be more burdensome than simply downloading daily step count from the Fitbit.com website. Although we were fortunate to be able to use an in-house platform (Eureka: https://info.eurekaplatform.org/), there are fee-based companies that offer such services. In addition, the M-M data and GMM model provide improved correlations with conventional measures and provide insight into how a patient spends their time in different activity levels. For instance, a larger “**μ3**” represents greater *average* step count; a smaller “**σ1**” denotes less variability; and “**π2**” denotes greater proportion of activities in the moderate range. These granular information combined with the level of disability (Tables 3A–C), provide a potential avenue for predictive algorithms and later, individualized rehabilitation plans.

**Table 3B:** Variance of physical activity level distribution per disability (EDSS) group (distribution of σ).

**EDSS group [*N*]**	**Disability level**	**Low variance σ_1_ (SD)**	**Moderate variance σ_2_ (SD)**	**High variance σ_3_ (SD)**
0.0–3.5 [36]	No “walking” disability	27.18 (9.99)	178.74 (78.72)	394.38 (191.20)
4.0–5.5 [26]	Walking disability present	23.67 (13.06)	152.10 (122.27)	487.65 (772.72)
6.0 [18]	Needs a cane to ambulate	17.31 (8.25)	83.20 (43.58)	362.86 (159.58)
6.5 [14]	Needs 2 canes or a walker to ambulate	10.47 (4.26)	58.32 (21.10)	294.96 (196.31)

*Each participant was fit individually, and the variance of each physical activity type was extracted for the group distribution. Greater levels of disability demonstrated smaller variance when compared with people characterized with lower disability scores*.

**Table 3C:** Proportion of physical activity level distribution per disability (EDSS) group (distribution of π).

**EDSS group [*N*]**	**Disability level**	**Low π_1_ (SD)**	**Moderate π_2_ (SD)**	**High π_3_ (SD)**
0.0–3.5 [36]	No “walking” disability	0.47 (0.07)	0.37 (0.04)	0.15 (0.06)
4.0–5.5 [26]	Walking disability present	0.51 (0.05)	0.36 (0.03)	0.13 (0.05)
6.0 [18]	Needs a cane to ambulate	0.55 (0.05)	0.34 (0.04)	0.11 (0.04)
6.5 [14]	Needs 2 canes or a walker to ambulate	0.61 (0.06)	0.31 (0.07)	0.09 (0.03)

*Each participant was fit individually, and the proportion of physical activity was extracted for the group distribution. The greater the level of disability (i.e. EDSS = 6.5) the higher proportion of lower levels of physical activity (**π**_1_) recorded, and the lower disability (i.e. EDSS = 0.0–3.5) the greater proportion of moderate (**π**_2_) and high physical activity (**π**_3_) levels recorded (Kruskal-Wallis Test: p = 0.0001)*.

*EDSS, Expanded Disability Status Score; N, sample size in each group; SD, standard deviation; μ1, mean steps - low physical activity; μ2, mean steps - moderate physical activity; μ3, mean steps - “high” (relatively) physical activity, Low variance, **σ**_**1**_; moderate variance, σ_**2**_ and high variance, **σ**_**3**_, **π**_1_, Low proportion of physical activity; **π**_2_, moderate proportion of physical activity; π_3_, high proportion of physical activity*.

Moderate-to-Vigorous activity equates with our π**2**, and has frequently been cited as the benchmark for determining optimal physical activity in MS and the general population. People needing double support to ambulate (EDSS = 6.5) tended to spend a larger proportion their activity in **π_1_** (corresponding to low levels of activity or sedentarism). On the other hand, people with lower disability (EDSS = 0.0–3.5) presented with a greater proportion in **π_2_** and **π_3_** (corresponding with more moderate and higher activity levels). Therefore, it may be more clinically useful to evaluate π**1**, and π**3** (the time spent in low levels and very high levels of activity) when assessing an individual patient's activity level and subsequent risk factors or rehabilitation needs. For example, understanding fluctuations in activity over the day to better personalize when to focus rehabilitation interventions and on what (intensity and duration of activity). Research awareness already shifted toward investigating the effect of sedentary time on health and disease (25–27). Higher physical activity has been associated with beneficial changes in the brain and spinal cord, as well with decreased levels of disability (28, 29). However, this area of investigation is in its infancy and the underlying mechanism of action is not yet understood. Methods presented in this paper could build on and support these lines of investigation – with aims at promoting greater overall wellness, and potentially delay disease progression in people with MS (29–32).

This model will be used as a framework to predict disease progression over the longer term (>2 years) and to develop further descriptive metrics for activity. How time spent at various activity levels is associated with MS disability over time – i.e., temporal validation of the Gaussian parameters for prediction on disability progression – remains to be determined. Models including associations of fall-risk prediction in people with MS would also be highly clinically valuable.

## Conclusion

Results from this analysis favor a 3 compartment GMM as the best probabilistic model to characterize dynamic ambulatory activity in people with MS with a wide range of disability. As compared to STEPS as a sole outcome, this method demonstrated stronger associations with conventional clinic-based and patient-reported outcomes. To unearth the full potential of this method, additional longitudinal exploration of predictive value is required.

## Data Availability Statement

The raw data supporting the conclusions of this article will be made available by the authors, without undue reservation.

## Ethics Statement

The studies involving human participants were reviewed and approved by UCSF Human Research Protection Program, Box 1288 490 Illinois Street, Floor 6 San Francisco, CA 94143. The patients/participants provided their written informed consent to participate in this study.

## Author Contributions

VB and MW: design and conceptualized study, analyzed the data, and drafted the manuscript for intellectual content. ZX and AA: analyzed the data and drafted the manuscript for intellectual content. RB, MP, GM, JO, and BC: interpreted the data and revised the manuscript for intellectual content. JG: design and conceptualized study, interpreted the data, and revised the manuscript for intellectual content. RH: design and conceptualized study, analyzed and interpreted the data, and revised the manuscript for intellectual content. All authors contributed to the article and approved the submitted version.

## Conflict of Interest

The authors declare that the research was conducted in the absence of any commercial or financial relationships that could be construed as a potential conflict of interest.

## Publisher's Note

All claims expressed in this article are solely those of the authors and do not necessarily represent those of their affiliated organizations, or those of the publisher, the editors and the reviewers. Any product that may be evaluated in this article, or claim that may be made by its manufacturer, is not guaranteed or endorsed by the publisher.
