# Supplementary material for: Making Every Step Count: Minute-by-Minute Characterization of Step Counts Augments Remote Activity Monitoring in People With Multiple Sclerosis
Source: Front Neurol. 2022 May 23;13:860008. doi: 10.3389/fneur.2022.860008 (PMC9167929; doi:10.3389/fneur.2022.860008)
Supplement: Supplementary file 1 [file Data_Sheet_1.PDF]

Supplemental Table 1. Comparison of Distribution Models

Distribution	AICc	BIC	-2*LogLikelihood
SHASH	4110555.5	4110599.9	4110547.5
Normal 3 Mixture (GMM)	4125308.1	4125397.0	4125292.1
Lognormal	4137243.0	4137265.2	4137239.0
Gamma	4184577.3	4184599.6	4184573.3
Negative Binomial	4197423.2	4197445.4	4197419.2
Weibull	4200194.6	4200216.8	4200190.6
Johnson Sb	4208862.0	4208906.5	4208854.0
Exponential	4219832.5	4219843.7	4219830.5
Normal 2 Mixture	4221148.3	4221203.8	4221138.3
Student's t	4421390.4	4421423.7	4421384.4
Cauchy	4443689.5	4443711.7	4443685.5
Normal	4580048.1	4580070.3	4580044.1
Poisson	11875951.0	11875963.0	11875949.0

Legend: AIC = Akaike's Information Criteria, BIC = Bayesian Information Criteria, SHASH = Sinh-Arcsinh distribution.

This Table illustrates the comparisons between distribution models.

We attempted multiple statistical models including poison, negative binomial, 2-Gaussian mixture model (GMM2), 3-Gaussian mixture model (GMM3), exponential, lognormal, SHASH and normall: both poisson and negative binomial come as nature choices for modeling count data. Exponential is the continuous analog of poisson and therefore serves as potential continuous alternative along with lognormal. When plotting the step count distributions, we observe clusters activities at median and high step counts regions and therefore we decide to attempt mixture distributions like GMM2 and GMM3 in hope to characterize the different clusters.

AICc and BIC are chosen to measure the goodness of fit of each model because they measure the likelihood of data given each model while putting a penalty on complex models.

Although the SHASH slightly outperformed the GMM3 for the pooled M-M step data with all subjects, when fit for individual subjects, the SHASH only outperformed the GMM3 on distributions without concentrations for higher step rates (higher intensity activity levels). Most participants’ activity was in the lower intensity range; hence, the pooled fit (SHASH) favored fitting the bulk of the activity.
